# Comparison of serum SDMA and creatinine as a biomarker for the detection of meloxicam-induced kidney injury in cats

**DOI:** 10.3389/fvets.2024.1395505

**Published:** 2024-05-15

**Authors:** Matthew K. Wun, Liam E. Broughton-Neiswanger, Nicolas F. Villarino

**Affiliations:** ^1^Department of Veterinary Clinical Sciences, College of Veterinary Medicine, Washington State University, Pullman, WA, United States; ^2^Department of Veterinary Microbiology and Pathology, College of Veterinary Medicine, Washington State University, Pullman, WA, United States

**Keywords:** symmetric dimethylarginine, acute kidney injury, feline, renal, biomarker

## Abstract

**Introduction:**

Serum symmetric dimethylarginine (SDMA) and creatinine are commonly used biomarkers of renal function in cats. We hypothesize that the serum analytes creatinine and SDMA are equally effective at detecting impaired renal function caused by meloxicam-induced renal injury in cats. Our primary objective was to compare serum concentrations of SDMA and creatinine in cats before, during, and after induction of renal injury from repeated dosages of meloxicam in the context of a small pilot study.

**Methods:**

This follow-up study results from data collected in a well-controlled study that included 12 healthy female adult purpose-bred cats. Cats in the treatment group received meloxicam 0.3 mg/kg subcutaneously (SC) every 24 h for 31 days. Cats in the control group received saline (0.1 mL SC). Renal injury was defined as the presence of tubular damage, basement membrane damage, and/or interstitial inflammation in histological sections of kidney tissue. Serum creatinine and SDMA concentration were measured every 4 days.

**Results:**

In the control group, no cats developed renal azotemia. In the treatment group, four out of six cats developed elevated serum creatinine and histopathological evidence of renal injury. Three of these cats developed an elevation in serum SDMA. The time to the development of renal azotemia using serum creatinine or SDMA was not significantly different (*p* > 0.05).

**Discussion:**

In this pilot study, there was no evidence that serum SDMA was superior to serum creatinine at detecting impaired renal function caused by meloxicam-induced renal injury in cats.

## 1 Introduction

Serum creatinine and symmetric dimethylarginine (SDMA) are currently the most commonly used analytes to estimate renal function in feline practice (1). Both are biomarkers of glomerular filtration rate (GFR). The coefficient of determination between creatinine and SDMA with GFR is reported to be 0.81 and 0.82, respectively (2), and the reported correlation of the two biomarkers with GFR are similar (3–5). Their relationship with GFR is exponential, requiring a substantial reduction in GFR before elevated serum values are observed (1). This presents a problem for the early detection of acute kidney injury (AKI) and chronic kidney disease (CKD), when medical interventions may be most effective.

Recent guidelines published by the International Renal Interest Society state that “SDMA appears to be a more sensitive indicator of early stage CKD in the dog and cat” (6) and “that compared with serum creatinine, SDMA can be a more sensitive renal function biomarker and provides additional information when used together with serum creatinine” (7). In cats, the evidence for these statements is derived from a 2014 study which found that the upper limit of the reference interval (RI) for SDMA (14 μg/dL) corresponded to a GFR of approximately 24% lower than the median GFR of healthy cats, with a sensitivity and specificity of 100 and 91%, respectively, for the detection of a 30% decrease from median GFR (3). However, the use of 14 μg/dL as the upper limit of the RI has recently been associated with false positive results for renal dysfunction (specificity 75%), with a higher cut-off value of 18 μg/dL recommended (5). In addition, this study found SDMA was not superior to creatinine in the detection of mild (GFR < borderline GFR cut-off of 1.7 mL/[min kg]) or obvious (GFR < low GFR cut-off of 1.2 mL/[min kg]) kidney dysfunction (5). As such, the proposed benefit of SDMA over creatinine remains unclear based on these conflicting scientific publications.

Meloxicam is a COX-2 preferential non-steroidal anti-inflammatory drug (NSAID) associated with the development of acute kidney injury in cats (8, 9). We hypothesize that the serum analytes creatinine and SDMA are equally effective at detecting impaired renal function caused by meloxicam-induced renal injury in cats. The primary objective of this pilot study was to compare serum concentrations of SDMA and creatinine in healthy adult purpose-bred cats, before, during, and after induction of renal injury from repeated dosing of meloxicam. Data collected in a prior well-controlled terminal metabolomics study (10) were used to address this objective.

## 2 Materials and methods

Data were collected from records of a previous research project completed at Washington State University (WSU) (WSU IACUC approved protocol# 4915) (10).

### 2.1 Study population, inclusion criteria and husbandry

The study population and experimental design have been described previously (10). Briefly, 12 female clinically healthy intact adult (1–1.5 years old) purpose-bred cats (2.5–3.8 kg) were obtained from a USDA-licensed commercial breeder (Nutrition and Pet Care Center, University of California Davis, Davis, United States).

Cats were acclimated to the new housing environment at least 10 days before beginning the study. Cats were housed separately in cages 49″ wide, 37″ tall, and 38″ deep. The room was temperature (21–23°C), humidity (25–35%), and 12-h light/dark cycle controlled. Throughout the study, the cats had free access to drinking water and food (Purina Cat Chow Indoor Formula). Each day, water, food, and litter were changed, and cages cleaned. The cats were examined at least twice daily during the entire study to rule out possible health problems. The cats’ estrus cycle was not evaluated objectively other than by monitoring behavior during the study. Following the acclimation period, vascular access ports (VAP, petite size) (Le Port Companion Port, Norfolk Vet, Skokie, IL, United States) were implanted in the jugular at least 7 days before starting the administration of the treatments following standard procedures as recommended by the manufacturer. The VAPs were maintained following the manufacturer’s recommendations.

### 2.2 Study design

**Figure 1:**
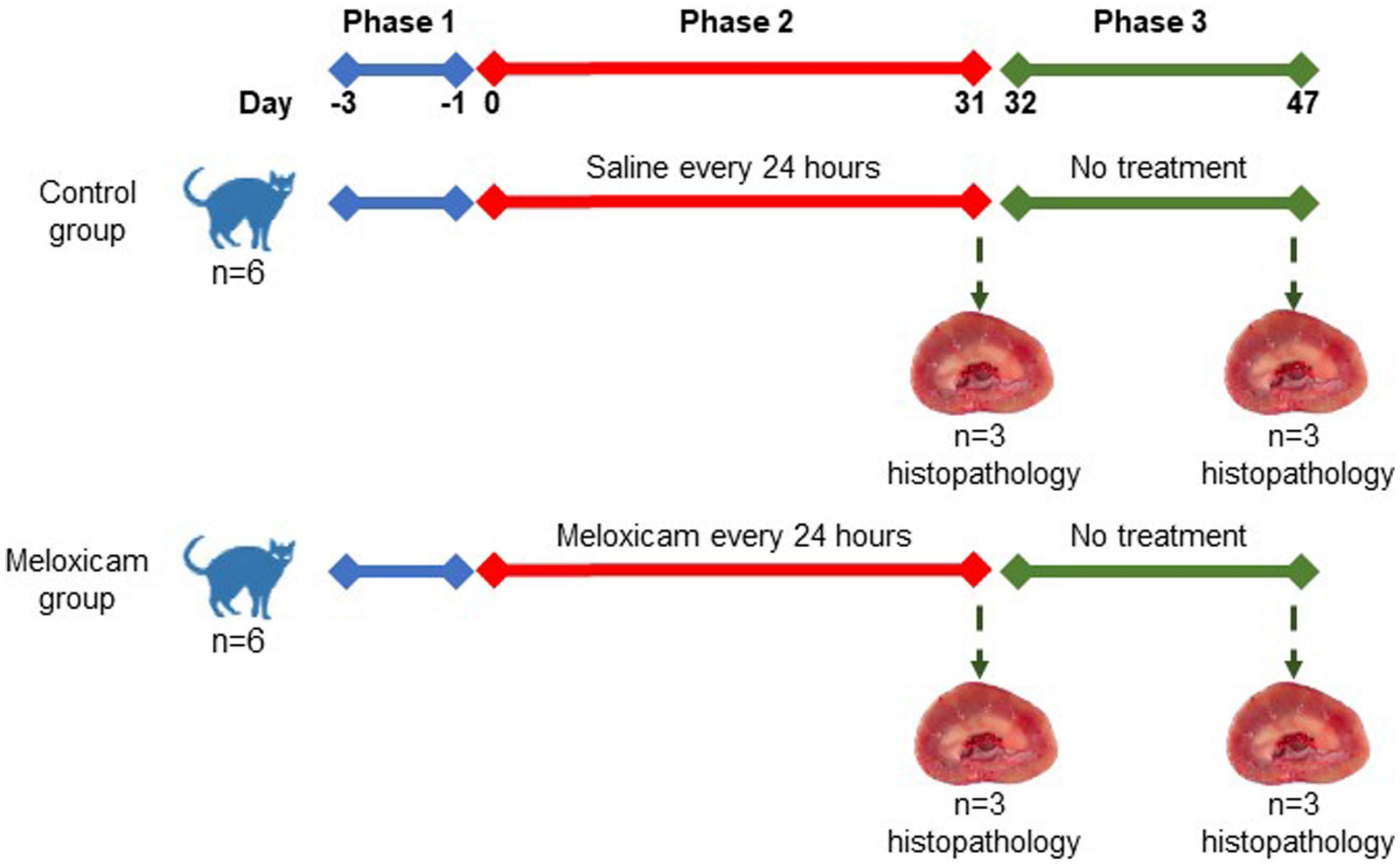
Schematic showing the experimental design used in this experiment.

In a controlled experimental design, cats were randomly allocated to four experimental groups: the (i) long-term (*n* = 3) and (ii) short-term (*n* = 3) control groups, and the (iii) long-term (*n* = 3), and (iv) short-term (*n* = 3) meloxicam groups. Randomization of the treatments was done using the RandomizeR package in R. The experiment comprised three consecutive phases, as depicted in Figure 1. Phase one lasted 3 days (day-3 to day 0), during which all cats were treated with 0.1 mL/kg body weight of saline subcutaneously (SC) every 24 h. During phase two of the experiment (starting day 0), cats in the meloxicam group were treated SC with meloxicam at a dosage of 0.3 mg/kg body weight (equivalent to 0.1 mL/kg) (Metacam^®^ injectable, Boehringer Ingelheim Vetmedica, Inc.) every 24 h for 31 days. Cats in the control group received a 0.1 mL/kg body weight saline SC every 24 h for 31 days. At the end of phase two, cats in the short-term control and meloxicam groups were euthanized within 24 h after the last treatment. During phase three, cats in the long-term control (*n* = 3) and meloxicam (*n* = 3) groups were monitored for 16 days (days 32–47), then euthanized with an IV overdose of pentobarbital (Beuthanasia-D, Intervet/Merck Animal Health, Giralda Farms, Madison, NJ).

Cats in the control group remained clinically healthy for the duration of the study, although one cat vomited once on day 4 after saline administration. In the meloxicam groups, the cats’ body weights and condition scores were relatively stable, except for one cat whose body weight was reduced by ~7%, likely due to a decrease in food intake. During phases two and three, five out of six cats in the meloxicam group vomited 2 to 15 times but no more than once a day. However, their food intake, body weight, and condition scores were consistent with pre-treatment values.

Tissues from both kidneys of all cats were collected postmortem. Immediately upon collection, tissue samples were preserved in 10% neutral buffered formalin. Replicate sections of each kidney were cut at 5 μm thickness and stained with hematoxylin and eosin for overall histologic grading and Masson’s trichrome for evaluation of fibrosis. Additional sections were cut at 3 μm thickness and stained with periodic acid-methenamine silver to assess the basement membrane of tubules and glomeruli. Tissues were evaluated for a series of semi-quantitative histologic features by a single, blinded, board-certified veterinary pathologist. Histologic features evaluated include cortical and corticomedullary tubular damage, basement membrane integrity, cortical fibrosis, medullary fibrosis, and interstitial inflammation. Tubular damage was defined as the presence of at least one or more of the following histologic features: epithelial cell necrosis, regeneration, degeneration, attenuation or karyomegaly, and tubular dilation. Fibrosis was defined as the expansion of the interstitium with trichrome-confirmed collagenous matrix in association with adjacent tubular injury. Semi-quantitative histologic features were scored as follows for all individual kidneys: 0 = no change to <1%, 1 = 1–25%, 2 = 26–50%, 3 = 51–75%, 4 = 76–100% of examined fields affected. A total of at least ten 400x high power fields were evaluated for the aforementioned histologic changes to produce the semi-quantitative score for each category, with 4 being the highest score per feature. In the meloxicam-treated groups, cats were determined to have renal injury if at least one kidney had a score of ≥1 for tubular damage, basement membrane damage, and/or interstitial inflammation (11). For data analysis, these cats were allocated to the meloxicam-treated renal injury group. Cats in the short and long-term control groups were allocated to a combined control group.

#### 2.2.1 Blood and urine sampling to determine SDMA, creatinine, and urine protein and creatinine concentration

Sampling times and procedures have been described previously (10) and are briefly summarized below.

#### 2.2.2 Sampling times

Blood and urine samples were collected during phase one on day-3 and immediately prior to phase two on day 0; during phase two, samples were collected on days 4, 9, 13, 17, 23, 26, and 31; in phase three, samples were collected on days 34, 40, and 47.

#### 2.2.3 Sampling procedures

The cats had no access to food for 8 h prior to blood and urine sampling. Blood samples were collected from a vascular access port (VAP) prior to treatment administration (which took place at 6 p.m. ± 1 h). Prior to sample collection, the VAP locking solution was aspirated and discarded. Blood (1.2 mL) was collected into tubes containing clot activator using sterile Huber needles. Immediately upon blood sample collection, serum was obtained by centrifugation (1800 x g for 8 min) and frozen. At the start of phase one, an additional 1.2 mL of blood was collected into an EDTA tube. Following blood collection, VAPs were flushed using heparinized saline solution (100 I.U./mL, 0.7 mL at each sampling time). Urine samples were collected immediately after blood sampling by ultrasound-guided cystocentesis following standard procedures. Upon urine collection, urine samples were centrifuged at 1800 x *g* for 8 min, and the supernatant was aliquoted and stored at-80°C until analysis. The cats had free access to drinking water throughout the study.

#### 2.2.4 Determination of serum SDMA and creatinine, and urinalysis

Frozen serum samples were submitted to IDEXX Laboratories (transported on dry ice within 24 h of collection) for serum biochemistry profiles (Chem 10 with IDEXX SDMA Test) and urinalysis. Samples were processed within 24 h after shipping. Azotemia was considered when serum creatinine concentration was ≥1.6 mg/dL, as per IRIS AKI guidelines (12). Two different upper limits of the RI were used for SDMA; 14 μg/dL [per IDEXX (13)] and 18 μg/dL (as recommended by Brans et al. (5) to reduce the likelihood of a false positive result for renal dysfunction). Renal azotemia was defined as serum creatinine and/or SDMA above the upper limit of the reference interval (RI) combined with a urine specific gravity ≤1.035 (14).

### 2.3 Statistical analysis

#### 2.3.1 Descriptive statistical analysis

Due to the small sample size, individual animal data for serum SDMA and creatinine are reported. Data distributions were tested using the D’Agostino and Pearson omnibus normality test and the Shapiro–Wilk test. Semi-quantitative histologic scores are reported as median (range) for the control and renal injury groups.

#### 2.3.2 Comparative statistical analysis

The area under the curve (AUC) (mean, standard error) for the serum concentration of SDMA and creatinine versus time curve for phase two (AUC_0-31 days_) and phase three (AUC_31-47 days_) for the meloxicam-treated renal injury and control groups were calculated for each individual by the trapezoidal method (15). The AUC_0-31 days_ and AUC_31-47 days_ for creatinine and SDMA for the two groups were compared statistically using an unpaired, 2-tailed t-test. Repeated measures correlation between serum SDMA and creatinine concentrations from the renal injury and control groups were calculated to determine if these two biomarkers were linearly correlated. In the renal injury group, the time to the development of renal azotemia using creatinine or SDMA concentrations was estimated using Kaplan–Meier survival analysis, and the survival functions were compared using the log-rank test. The analysis was performed twice, using 14 μg/dL and 18 μg/dL as the upper limit of the RI for SDMA. The level of significance for the statistical comparisons was set at *p* ≤ 0.05. Descriptive, AUC, and Kaplan–Meier survival analyses were performed using Prism 9.5.0 (GraphPad Software, LLC). Repeated measures correlation analysis was performed using rmcorrShiny (16).

## 3 Results

### 3.1 Renal histopathology

**Figure 2:**
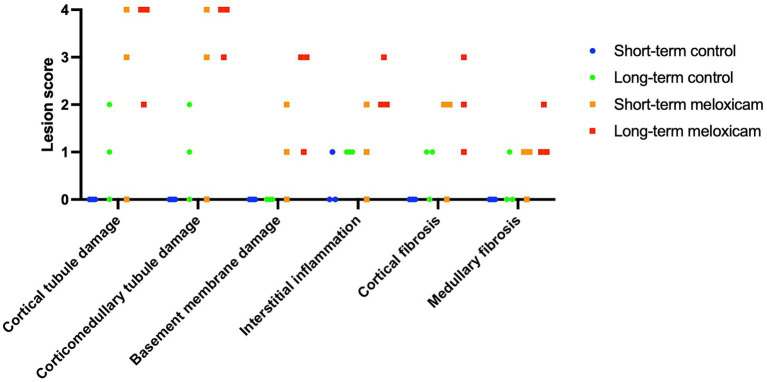
Semi-quantitative histological scores. The six data points for each group encompass three cats with scores for each individual kidney.

Renal histopathological findings are summarized in Figure 2 and Table 1. Two cats in the short-term meloxicam group did not have evidence of renal injury, did not develop renal azotemia during the study (maximum serum creatinine concentrations of 1.0 mg/dL and 1.1 mg/dL), and were excluded from further analysis. The four remaining cats in the meloxicam groups did have evidence of renal injury and were included in the meloxicam-treated renal injury group. Of the six cats in the control group, one cat had mild interstitial inflammation and mild–moderate cortical and corticomedullary tubular damage, and mild interstitial inflammation was present in two other cats (Figure 2).

**Table 1:** Semi-quantitative histologic scores are presented as median (range).

	Control group (*n* = 6)	Meloxicam-treated renal injury group (*n* = 4)
Cortical tubule damage	0 (0, 2)	2.5 (1, 4)
Corticomedullary tubule damage	0 (0, 3)	3 (1, 4)
Basement membrane damage	0 (0, 0)	1.5 (1, 3)
Interstitial inflammation	0 (0, 1)	1.5 (0, 3)
Cortical fibrosis	0 (0, 1)	2 (1, 3)
Medullary fibrosis	0 (0, 1)	1 (0, 3)

### 3.2 Changes in serum SDMA, creatinine, and urine specific gravity

**Figure 3:**
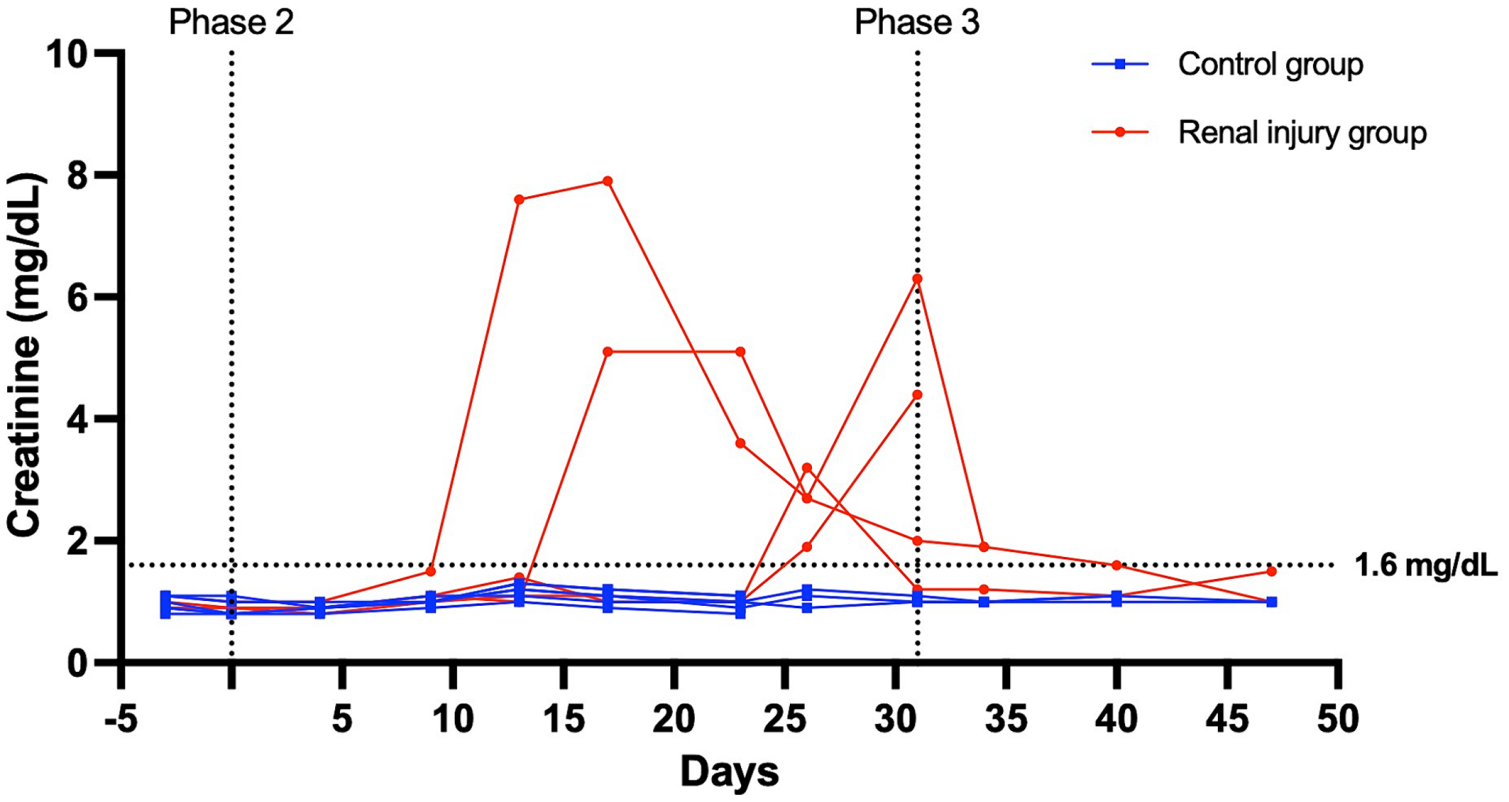
Serum creatinine versus time profile for six cats in the control group and four cats in the meloxicam-treated renal injury group. During phase 2 (days 0–31), cats in the renal injury group were administered meloxicam 0.3 mg/kg SC every 24 h, and cats in the control group were administered saline 0.1 mL/kg SC every 24 h. Cats were monitored during phase 3 (days 32–47). A serum creatinine ≥1.6 mg/dL was used to define azotemia.

**Figure 4:**
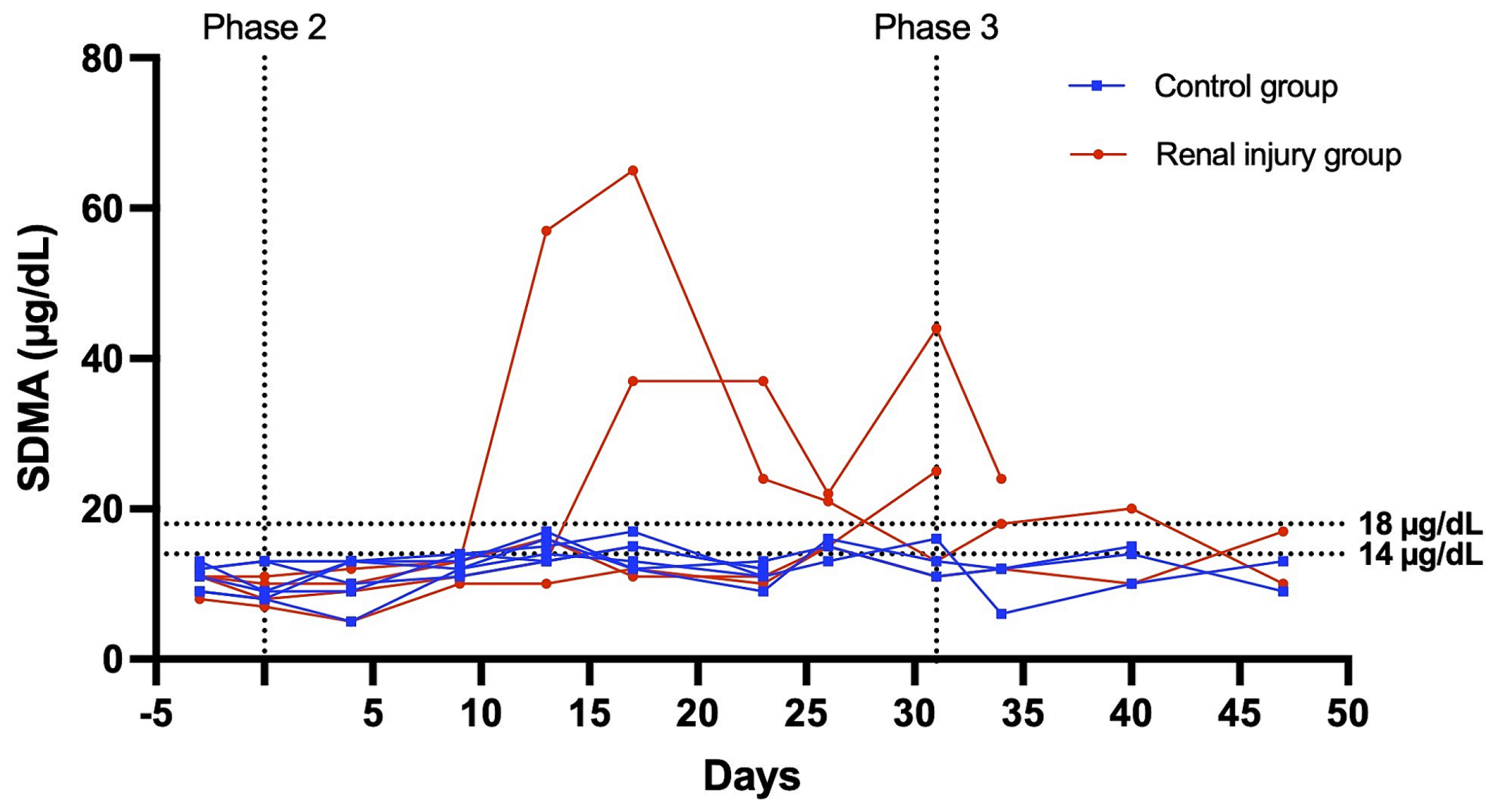
Serum SDMA versus time profile for six cats in the control group and four cats in the meloxicam-treated renal injury group. During phase 2 (days 0–31), cats in the renal injury group were administered meloxicam 0.3 mg/kg SC every 24 h, and cats in the control group were administered saline 0.1 mL/kg SC every 24 h. Cats were monitored during phase 3 (days 32–47). An SDMA ≥14 mg/dL and ≥ 18 mg/dL were used to define azotemia.

**Figure 5:**
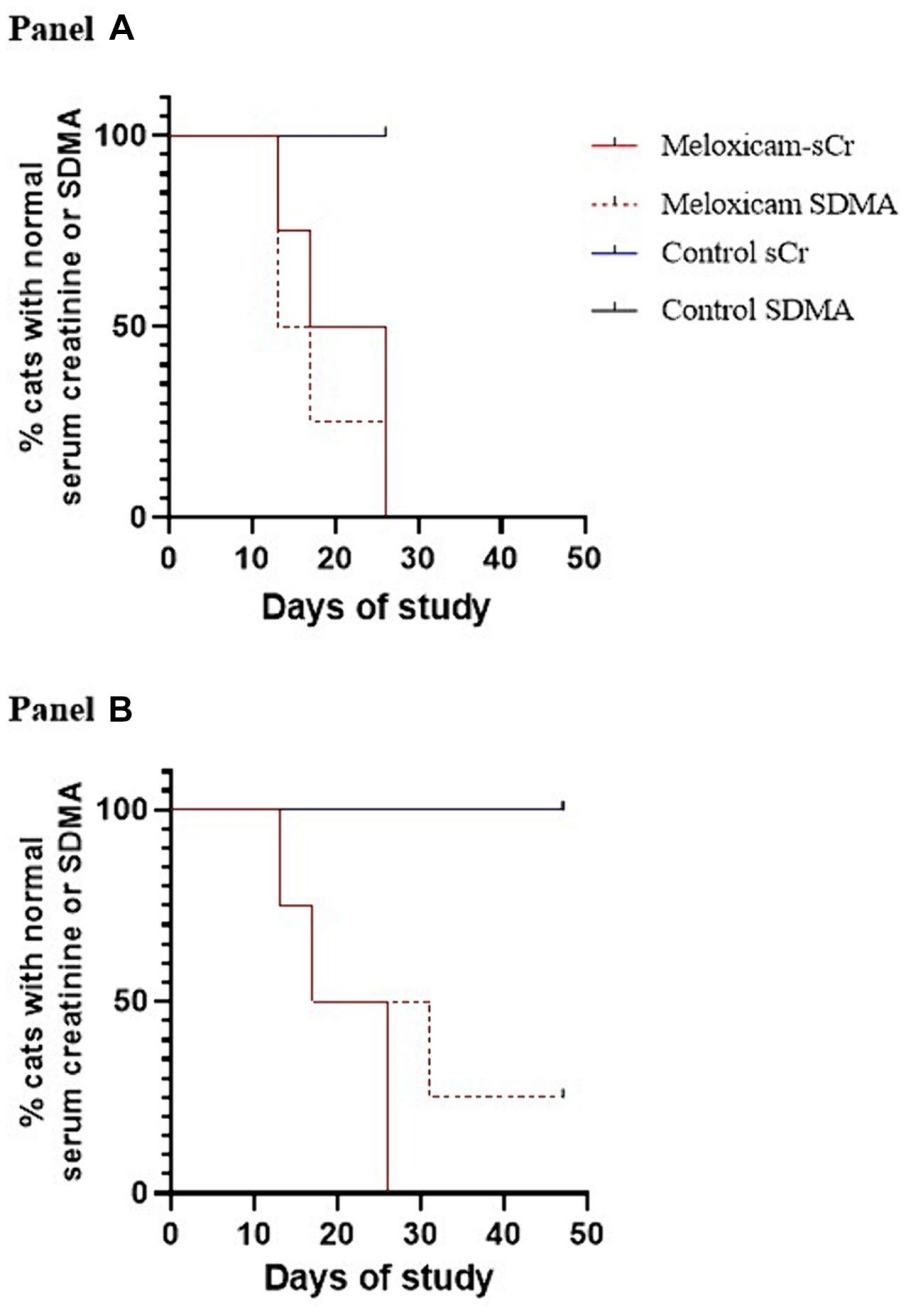
Kaplan–Meier survival curves depicting the time at which cats with meloxicam-induced renal injury developed azotemia using serum creatinine and SDMA (*n* = 3). Azotemia was defined as a serum creatinine ≥1.6 mg/dL, and a serum SDMA >14 mg/dL **(A)** or > 18 mg/dL **(B)**.

At the start of phase one, all cats had a normal physical exam, complete blood count, serum chemistry profile, and urinalysis, with specific gravities >1.055. All the data were normally distributed. The serum creatinine and SDMA concentration vs. time profiles for each cat are presented in Figures 3, 4. The AUC_0-31 days_ for creatinine in the control and meloxicam-treated renal injury groups was 31.74 (0.9014) and 72.39 (17.66), respectively (*p* = 0.0085). The AUC_0-31 days_ for SDMA in the control and renal injury groups was 395.9 (17.23) and 587.0 (126.0), respectively (*p =* 0.0823). The AUC_31-47 days_ for creatinine in the control and meloxicam-treated renal injury groups was 16.48 (0.2799) and 25.86 (4.262), respectively (*p* = 0.0454). The AUC_31-47 days_ for SDMA in the control and meloxicam-treated renal injury groups was 188.0 (19.90) and 260.6 (47.79), respectively (*p =* 0.1826). All cats that developed a serum creatinine and/or SDMA above the upper limit of the RI developed a concurrent reduction in urine specific gravity to ≤1.035. Serum creatinine and SDMA concentrations in the meloxicam-treated renal injury group were strongly linearly correlated (r_repeated measures_ = 0.96, *p* = <0.001), whereas the control groups moderately linearly correlated (r_repeated measures_ = 0.62, *p* = <0.001). In the meloxicam-treated renal injury group, the time to the development of renal azotemia using creatinine or SDMA was not significantly different using 14 μg/dL (median of 21.5 days and 15.0 days, respectively, *p* = >0.9999) or 18 μg/L (median 24.0 days, *p* = 0.8838) as the upper limit of the RI for SDMA (Figures 5A,B). No cats in the control group developed renal azotemia. Renal histopathology scores, creatinine, and SDMA AUC values for each cat in the meloxicam-treated renal injury group are presented in Supplementary Table S1.

## 4 Discussion

This pilot study compares the effect of repeated SC administration of 0.3 mg/kg of meloxicam daily on serum creatinine and SDMA. Our results provide insufficient evidence to reject our null hypothesis that serum creatinine and SDMA are equally effective at detecting impaired renal function caused by meloxicam-induced renal injury in cats.

One of the most revealing findings of this study is that there appears to be significant individual variability in how cats tolerate meloxicam. Two out of six cats in our study failed to develop renal injury despite receiving meloxicam 0.3 mg/kg SC every 24 h for 31 days. Similar variability was observed in meloxicam’s FDA New Animal Drug Application, which reports that four out of six cats had no histopathological evidence of renal injury after being administered meloxicam 0.3 mg/kg SC every 24 h for three days (17). Other recent evidence suggests that renal injury can occur in the absence of hemodynamic challenge in cats following a single SC injection at a dose ≤0.3 mg/kg (9) and after repeated doses (8), whereas other studies demonstrate the safety of meloxicam when given PO at a repeated low dose (0.01–0.03 mg/kg SID) (18–20). Factors explaining individual tolerance or susceptibility to meloxicam, and whether this interindividual variability is extended to other NSAIDs, remains unclear but deserves further research.

In the saline-treated control group, three cats had some subtle renal lesions of unknown origin but did not develop azotemia (Table 1; Figure 2), suggesting these renal lesions did not alter GFR sufficiently to alter serum creatinine and SDMA renal filtration. Four out of the six meloxicam-treated cats had widespread renal lesions. In all four of these cats, serum creatinine concentration increased above the IRIS reference interval. Similarly, all cats had a serum SDMA concentration above the IDEXX reference interval (14 μg/dL). However, in one cat, serum SDMA did not exceed the 18 μg/dL cut-off recommended by Brans et al. (5). Elevations of both biomarkers in the meloxicam-treated renal injury group presumably reflect functional changes induced by meloxicam resulting in a reduction in GFR (21). However, GFR was not measured directly in this study. The renal lesions observed may lead to proximal luminal obstruction and backflow of filtrate across injured proximal tubular cells, resulting in a decrease in GFR (22).

During stages two and three of this study, meloxicam-treated cats with renal injury had significantly higher serum creatinine concentrations than control cats (*p =* 0.0085 and 0.0454, respectively) (Figure 3). However, serum SDMA in the meloxicam-treated renal injury group was not significantly higher than the control group in either phase of the study, despite good correlation with creatinine (*r* = 0.9524 and *r* = 0.8410 in the meloxicam-treated renal injury and control groups, respectively) (23, 24).

Another relevant result of this study is the substantial lag time between the induction of renal injury and the development of renal azotemia. In the meloxicam-induced renal injury group, the time to develop renal azotemia using creatinine or SDMA was not different (*p* > 0.05), despite using 14 μg/dL as the upper limit of the RI for SDMA as specified by IDEXX. It is unknown when the renal changes and GFR start to decline during the treatment course. However, we were expecting that elevation in these biomarkers would occur sooner, considering that administration of meloxicam 0.3 mg/kg subcutaneously every 24 h for 3 days caused dilated cortical tubules and interstitial inflammatory cell infiltration in some cats (17). Notably, in our study, creatinine and SDMA were also unable to detect the presence of subtle renal histopathological lesions in three of our control cats. Considering the potentially extended lag time (interval between abnormal GFR and abnormal serum creatinine/SDMA) and renal lesions observed in this study, urinary markers of epithelial cell stress (such as kidney injury molecule-1), and small molecules excreted in urine such as taurine, tryptophan, tyrosine, lyxitol, pseudouridine, xylitol, threonic acid (10) and cystatin B (23) could be more sensitive than GFR biomarkers to detect early renal changes induced by meloxicam. Our results suggest that these markers of kidney injury should be the focus of future research in this area, and further terminal studies investigating changes in GFR biomarkers may not be ethically justifiable.

Our findings are consistent with those reported by Brans et al. (5), who retrospectively measured serum creatinine, SDMA, and GFR in 17 cats with CKD, 15 cats with diabetes mellitus, and 17 healthy cats. Receiver operating characteristic (ROC) analysis of this dataset showed that SDMA was not superior to creatinine in the detection of mild or obvious renal dysfunction (5). This, and the results of our study, do not support current IDEXX marketing materials that *“SDMA can detect mild to moderate function loss that creatinine misses*” (24). In cats, this claim appears to be derived from a single, retrospective study evaluating serum creatinine, SDMA, and GFR in 21 cats with CKD and 21 healthy, geriatric cats (3). Unfortunately, this study has significant limitations, including a lack of information on sample selection criteria (cats were selected from a colony of over 400) and ROC analysis of SDMA concentrations, with conclusions instead drawn from unspecified, unvalidated linear and nonlinear models (3). In addition, 2.1 mg/dL was used as the upper limit of normal for creatinine, as opposed to <1.6 mg/dL specified by IRIS (6). As such, the reported sensitivity of serum creatinine for the detection of CKD in this study is likely to be lower than that obtained had IRIS guidelines been followed. Another study documenting SDMA and creatinine concentrations in a cohort of dogs and cats with naturally occurring renal disease used an even higher upper limit of normal serum creatinine (2.3 mg/dL) (25).

The main limitation of this study is its small sample size and the associated risk of type II error. No *a priori* power analysis was performed, and as such, it is unclear whether the time to abnormal biomarker concentration comparison reflects a truly negative or underpowered study. Another limitation of this study is the lack of renal tissue samples and renal histopathology studies before and during the administration of the treatments, therefore it is unknown at what time point the renal microscopic lesions occurred. Pre-treatment biopsies could provide valuable information as part of the inclusion criteria of animals and should be considered in future studies.

The extent to which our findings can be extrapolated to cats with naturally occurring renal disease is open to contention. The exact mechanism through which meloxicam induced renal injury and elevated serum creatinine and SDMA in some cats in our study is unclear (26, 27). It is doubtful that our, or any, experimental model of feline renal disease can recapitulate all aspects of naturally occurring AKI or CKD, given the range of possible etiologies that oftentimes are unknown (28, 29). On the other hand, cats in our renal injury group had renal histopathological changes comparable to those seen with naturally occurring CKD and AKI (11, 30, 31), and the clinical course of meloxicam-associated AKI is comparable to other causes of AKI in cats (9, 28).

To our knowledge, this is the only prospective study evaluating serum creatinine and SDMA concentrations prior to, during, and following the induction of renal injury in cats. Our findings, in the context of the current literature and the experimental conditions, provide no evidence to suggest that serum SDMA is superior to serum creatinine at detecting impaired renal function caused by meloxicam-induced renal injury in cats, and highlight the need for the discovery and validation of biomarkers for detecting early kidney injury.

## Data availability statement

The raw data supporting the conclusions of this article will be made available by the authors, without undue reservation.

## Ethics statement

The animal study was approved by IACCUC Washington State University. The study was conducted in accordance with the local legislation and institutional requirements.

## Author contributions

MW: Data curation, Formal analysis, Investigation, Methodology, Writing – original draft, Writing – review & editing. LB-N: Methodology, Writing – review & editing. NV: Conceptualization, Data curation, Formal analysis, Funding acquisition, Investigation, Methodology, Project administration, Resources, Software, Supervision, Validation, Visualization, Writing – original draft, Writing – review & editing.
