# Supplementary material for: Comparison of serum SDMA and creatinine as a biomarker for the detection of meloxicam-induced kidney injury in cats
Source: Front Vet Sci. 2024 May 15;11:1395505. doi: 10.3389/fvets.2024.1395505 (PMC11133641; doi:10.3389/fvets.2024.1395505)
Supplement: Supplementary file 1 [file Table_1.docx]

Supplementary Table 1. Semi-quantitative histologic scores and area under the concentration vs time profile of serum creatinine (SCr) and SDMA

| Cat | **Group** | Cortical tubule damage | Corticomedullary tubule damage | Basement membrane damage | Interstitial inflammation | Cortical fibrosis | Medullary fibrosis | Phase 2  SCr (mg*h/dL) | Phase 2  SDMA AUC (µg*h/dL) | Phase 3 SCr AUC (µg*h/dL) | Phase 2 SDMA AUC (µg*h/dL) |
| --- | --- | --- | --- | --- | --- | --- | --- | --- | --- | --- | --- |
| 1 | Meloxicam-treated  renal injury | 3.5 | 3.5 | 1.5 | 1.5 | 2 | 1 | 42.9 | 349 | N/A | N/A |
| 2 | Meloxicam-treated  renal injury | 4 | 4 | 2 | 2.5 | 1.5 | 1 | 89.8 | 707.5 | 12.3 | 102 |
| 3 | Meloxicam-treated  renal injury | 2 | 3 | 2.5 | 1.5 | 3 | 2.5 | 115.2 | 901 | 25.45 | 265.5 |
| 4 | Meloxicam -treated  renal injury | 1 | 1 | 1 | 0.5 | 1.5 | 0.5 | 41.7 | 390.5 | 19.6 | 195 |
| 5 | Meloxicam-treated  no renal injury | 0 | 0 | 0 | 0 | 0 | 0 | 26.15 | 319.5 | N/A | N/A |
| 6 | Meloxicam-treated  no renal injury | 0 | 0 | 0 | 0 | 0.5 | 0.5 | 28.95 | 281.5 | N/A | N/A |
| 7 | Control | 0.5 | 0.5 | 0 | 1 | 0.5 | 0 | 30.6 | 398 | 9.3 | 118.5 |
| 8 | Control | 1.5 | 2.5 | 0 | 1 | 1 | 1 | 32.5 | 355 | 16.15 | 161.5 |
| 9 | Control | 0 | 0 | 0 | 0 | 0 | 0 | 31.4 | 400.5 | 16.65 | 193 |
| 10 | Control | 0 | 0 | 0 | 0.5 | 0 | 0 | 25.25 | 325.5 | N/A | N/A |
| 11 | Control | 0 | 0 | 0 | 0 | 0 | 0 | 25.5 | 283.5 | N/A | N/A |
| 12 | Control | 0 | 0 | 0 | 0 | 0 | 0 | 20.15 | 284.5 | N/A | N/A |
